# The relationships between the multidimensional planned behavior model, green brand awareness, green marketing activities, and purchase intention

**DOI:** 10.1002/brb3.3584

**Published:** 2024-06-14

**Authors:** Duygu Çinar Baltaci, Yakup Durmaz, Furkan Baltaci

**Affiliations:** ^1^ Department of Hair Care and Beauty Services, Vocational School of Social Science Gumushane University Gumushane Turkey; ^2^ Department of Marketing, Faculty of Economics, Administrative & Social Sciences Hasan Kalyoncu University Gaziantep Turkey; ^3^ Department of Tourism Management Hasan Kalyoncu University Gaziantep Turkey

**Keywords:** Generation Z, green awareness, green brand, green marketing, planned behavior, purchase intention

## Abstract

**Introduction:**

This study investigates the determinants impacting consumer purchasing behavior.

**Methods:**

Utilizing multidimensional planned behavior theory alongside measures of brand awareness and green brand awareness, this study examines the mediating role of multidimensional planned behavior theory. Empirical data were gathered through a survey conducted among Generation Z university students, yielding 638 responses. Analysis was performed on 590 valid responses.

**Results:**

Findings indicate that green marketing initiatives and green brand awareness positively influence consumers’ purchase intentions. Subdimensions of the theory of planned behavior (TPB), namely, attitude, subjective norms, and perceived behavioral control, serve to strengthen these relationships and mediate the interaction process.

**Conclusion:**

This study contributes novel insights to the burgeoning field of green marketing literature, offering a structural model for firms and policymakers. It suggests that companies can effectively engage in informal consumer education through green marketing efforts, thereby influencing consumers’ purchasing decisions via planned behavior. Moreover, such activities foster an increase in consumers’ green brand awareness, providing companies with an opportunity to promote conscious consumer behavior. The study's utilization of the TPB is both timely and original, particularly in its alignment with the United Nations 2030 Sustainable Development Goals.

## Graphical abstract

This study investigates the mediating role of green marketing activities in the effect of green brand awareness on consumer purchase intention. A survey was conducted with university students in the Generation Z consumer class for empirical research. A total of 638 feedbacks were provided in the online survey application. The analyses were conducted on 590 valid survey data.

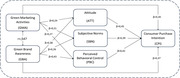

## 1 INTRODUCTION

The mechanization process initiated during the Industrial Revolution has positioned production as the central focus for all stakeholders (Alaloul et al., 2020). This revolutionary paradigm is predominantly anchored in developmental concepts and quantitative metrics such as output quantities, profits, and labor utilization, foregrounding the overall production process (Phuc et al., 2023). However, this production‐centric management approach has led to a significant oversight of other consequential issues, particularly environmental concerns (Patel & Mehta, 2023). Historically, environmental challenges were often perceived as inevitable byproducts of development that had to be endured (Baltaci, 2021).

The continual escalation of production volumes daily has exacerbated environmental issues, intensifying pressures and depleting natural resources to the point of straining carrying capacities (Khan et al., 2021). The escalation of these problems to a critical level has spurred the activation of solution mechanisms. A pivotal milestone along this trajectory was the publication of the report titled “The Limits of Growth” (Meadows et al., 1972), followed by the landmark report “Our Common Future” (Brundtland Report) issued by the UN Environment and Development Commission (UN, 1987). Sustainability has been posited as a fundamental and efficacious approach to addressing existing environmental challenges. The United Nations Millennium Development Goals and the subsequent 2030 Sustainable Development Goals have been established as ongoing processes to achieve these objectives (UN, 2024).

The heightened emphasis on environmental consciousness has also galvanized marketing researchers. Within the marketing literature, studies have emerged focusing on environmental sustainability, encompassing topics such as green consumer behavior (Cheung & To, 2019), responsible tourism behavior (Lin et al., 2022), green supply chains (Badi & Murtagh, 2019), and corporate carbon footprints (Freeman & Nunez, 2020). Presently, the advent of Industry 3.0 and its successor, Industry 4.0, represents pioneering endeavors spearheaded by the service, agricultural, and industrial sectors, aiming to minimize environmental impacts.

The efforts of businesses alone are not enough to achieve sustainability goals. Its stakeholders are required to make the same effort. Businesses have the opportunity to direct the behavior of their stakeholders through advertising and marketing activities (Alsharif et al., 2022). Most consumer behavior is unconscious and requires guidance (Ozkara & Bagozzi, 2021). Neuromarketing activities are important elements that guide and increase the awareness of both customers and intermediaries (Alsharif, Salleh, Hashem, et al., 2023). Consumers respond to companies’ green marketing communications and engage in green purchasing behavior (Correia et al., 2023). To increase consumers’ willingness to purchase green brands, organizations should improve their green brand knowledge by increasing consumers’ green brand image perception and trust (Tan et al., 2022). The green brand awareness of consumers is also effective in their green purchasing processes (Kamalanon et al., 2022). Green awareness or environmental knowledge is considered the antecedent of the green purchasing behavior process (Asif et al., 2023). The relationship between brand awareness, marketing activities, and purchasing behavior has also been proven in academic studies (Arya et al., 2024; Mehraj et al., 2023; Sharma, 2021; Singh et al., 2023; Watson et al., 2024). Namely, the messages chosen by advertisers directly affect consumers emotions, thoughts, attention, and memories. Studies conducted in this field, which represents neuromarketing, have revealed the close relationship between emotions and advertisements (Alsharif, Salleh, Alrawad, et al., 2023). Thus, brand awareness needs to be created to induce green purchasing behavior in potential consumers (Pratama et al., 2023; Tan et al., 2021). This process can be catalyzed by advertising and promotional activities, which are the attitude (ATT) formation step. It is also known that ATTs are important emotions that direct behavior (Ajzen et al., 2018). Internal and external stimuli lead consumers to have a positive or negative ATT (Kimiagari & Malafe, 2021). This is a cognitive process. Consumers’ awareness of brands points to the attitudinal process. Shaping the process with positive stimuli triggers purchasing behavior (Alsharif & Alharbi, 2022). There is a gap in studies testing the impact or mediating role of planned behavior in the ATT and behavior process. Knowing the impact of consumers’ planned behavior on their ATTs and behaviors will help companies. Thus, companies will have a clear recommendation on what to pay attention to in the process of creating brand awareness through advertising and promotional activities (Sun et. al., 2022).

The readiness of potential consumers is important in the success of the ATT development process. In this respect, Generation Z (GEN Z) has a great readiness potential (Wiedmer, 2015). GEN Z, born in 1995 and later, is digital natives who are intensely interested in technology. Mammadli (2023) stated that GEN Z is more sensitive to environmental problems, environmental protection, and pollution reduction. It has even revealed that its environmental concerns encourage sustainable consumption. Today, GEN Z has entered the business world and become an active buyer. Thus, GEN Z will be the direct addressee of the 2030 and 2050 sustainable development goals. For this reason, examining the purchasing behavior of GEN Z and determining the push and pull factors are important. It is known that the theory of planned behavior (TPB) plays a mediating role in explaining the green purchasing behavior of GEN Z (Pan et al., 2022).

This study approaches the literature from a holistic perspective and tries to explain consumers’ green purchasing tendencies through the extended TPB. The vast majority of GEN Z is now at the university level. For this reason, the future projection of the study is based on GEN Z. Thus, we aimed to add further detail to the study results. The results of this study will help businesses understand the behavior of GEN Z consumers, which is an expanding market. The results reveal the relationships between green brand awareness and green shining activities and between green brand awareness and consumer purchasing behavior with a structural model. Additionally, this study will prove the mediating effect of the TPB in the model and contribute to the expanding green marketing literature.

In the paper, green marketing activities were primarily examined. To achieve the aims of the study, we conducted an in‐depth literature review. In this way, we created the theoretical basis of the study. In this phase, we developed and conceptualized the research hypotheses. We found studies that focused on the relationships between our dependent, independent, and mediating variables. Thus, we contextualized the relationships between our variables and strengthened our hypotheses. We determined the most appropriate scales and population for our study. Afterward, we collected and analyzed our data. We tested the reliability and validity of our scales. For this, we used the Cronbach alpha coefficient and structural validity test (confirmatory factor analysis [CFA]). We used structural equation modeling (SEM) to test the relationships between variables. We also tested our hypotheses. In Section 4, we discussed our results with literature references. Thus, we proved the general validity of our hypotheses and results. After discussing the data obtained, the paper was finalized with the results, limitations, and recommendations sections. The fact that the mediation model has not been tested before is considered a significant risk of the study. However, based on the relevant literature, it was assumed that the awareness and readiness levels of GEN Z were high. These should be considered difficulties of the work.

## 2 LITERATURE REVIEW

### 2.1 Green marketing activities

Green marketing includes various activities, such as product differentiation, differences in the production workflow, changes in packaging, and differentiation of advertising activities (Tan et al., 2022). Similarly, Mehraj and colleagues (2023) and Welford (2000) defined green marketing as a management process that identifies, predicts, and satisfies the needs and desires of customers and society way profitably and sustainably. Companies support the green transformation to maintain their competitiveness (Padilla‐Lozano & Colazzo, 2021). This is possible by producing effective solutions to environmental problems. Companies achieve this by turning to ecologically safer products (Tsendsuren et al., 2021), recyclable and biodegradable packaging (Bhardwaj et al., 2020), better pollution control, and more energy efficient operations (Wang et al., 2023; Kotler & Armstrong, 1995). Environment‐friendly products aim to reduce negative impacts on the environment. It also provides significant improvements to the environment throughout its entire life cycle (Tan et al., 2022).

Green marketing is a sustainable marketing strategy. It focuses on profitably identifying and forecasting the needs of end customers and society. For this purpose, businesses create a holistic management process (Mehraj et al., 2023). Green marketing of goods or services covers the processes of design, production, packaging, labeling, marketing, use, and disposal (Correia et al., 2023).

In the marketing literature, the pioneers of environmental typologies focused on the general business environment rather than on a distinctly “green” marketing perspective (Zeithaml & Zeithaml, 1984). Similarly, McDaniel and Kolari (1987) and Walker and Ruekert (1987) designed their typologies for the general business environment based on Miles et al. (1978) classification of “reactor‐defender‐analyzer‐seeker.” According to these authors, this classification is a useful theoretical framework for analyzing the interactions of organizations with their environment and marketing strategies. Miles et al. (1978) classified firms according to adaptive decision models (reactors, defenders, and analyzers) and a more harmonious category (researchers). However, the “reactor” group should be left out of the continuum as it refers to organizations that have not set any specific strategies (Parnell & Wright, 1993). Dec Sundays “Defenders” have narrow product‐market areas; they are focused on maintaining their positions and do not tend to search outside these areas for new opportunities (Obel & Gurkov, 2023). Dec Sundays “analysts” focus on maintaining their positions in the main markets but also want to innovate at the margins, selectively searching for new product opportunities (Miles et al., 1978). Finally, “researchers,” on the other hand, want to access the widest Sunday possible by making consistent efforts to innovate and produce changes in their industry (Parnell & Wright, 1993). By following emerging environmental trends, they often measure newly formed behavioral patterns in front of consumers.

Consumers pay attention to green products to reduce negative environmental impacts. The pricing policies of companies positively affect the purchasing decision process of consumers (Nekmahmud & Fekete‐Farkas, 2020). The advertising strategies of companies support sustainable development. It affects consumers cognitively and directs their purchasing behavior (Dai & Sheng, 2022). Green advertisements increase consumers’ awareness of environment‐friendly products and induce purchasing intentions (Bi et al., 2023). There are studies examining the interaction process between green marketing activities and purchasing behavior within the framework of the TPB (Almrafee & Akaileh, 2023; Asif et al., 2023; Lin & Dong, 2023; Wongsaichia et al., 2022). Based on these empirical findings, the following hypotheses were established:

**H_1_**. Advertising activities have a positive and significant effect on ATT.
**H_2_**. Advertising activities have a positive and significant effect on subjective norms (SBNs).
**H_3_**. Advertising activities have a positive and significant effect on perceived behavioral control.
**H_10_**. Advertising activities have a positive and significant effect on consumer purchase intention.

### 2.2 Green brand awareness

Brands are meaningful systems that contain values, ideas, associations, feelings, and emotions that form a more or less consistent identity (Ko et al., 2019). Brands allow for the differentiation and protection of products from similar products of competitors. When examining a brand, six basic dimensions are the focus. These are (Kotler & Keller, 2006) as follows:

- Attributes: The brand covers a certain set of characteristics;
- Benefits: The qualities of the brand should translate into functional and emotional benefits that are valued by consumers;
- Values: The brand conveys something about the values of the company;
- Culture: The brand can represent a specific cultural expression;
- Personality: The brand can convey a certain personality, which is gradually built up through marketing communications; this personality can reflect what kind of person the brand would be if it were human;
- User definition: The brand recommends the type of consumer who buys or uses it.

Within brands, a green brand is defined as a specific group of brand characteristics and benefits related to minimizing the environmental impact of the brand and its perception as environmentally healthy (Asif et al., 2023). Therefore, a green brand should benefit consumers who are more sensitive to the environment. To be successful, a green brand must offer a significant eco advantage over other brands and be aimed at consumers who are willing to value environmental problems (Kamalanon et al., 2022). This means that a green brand should communicate with its target audience as consumers’ beliefs about the good ecological performance of the brand lead to a positive ATT toward that brand (Kinnunen et al., 2022).

Brand awareness is a condition in which customers recognize the brand of a product and connect to certain product categories correctly (Alamsyah & Febriani, 2020). However, it is how consumers associate the brand with a specific product that they intend to have. Brand awareness is indispensable for the emergence of the communication process, that is, for memorable awareness (Gómez‐Rico et al., 2023). Effective marketing communication channels such as television, mobile devices, and online channels help reduce product evaluation and selection risk while ensuring product quality and reliability (Pancić et al., 2023). Brand awareness significantly affects the consumer decision‐making process when consumers often use it as a heuristic decision that benefits the management of customer‐based brand value (Tan et al., 2021).

Green brand awareness is characterized by the possibility of identifying and remembering (RM) the characteristics of a brand that is dedicated to protecting the environment (Rahmadhani & Widodo, 2023). Green brand awareness, brand reputation, policies, and the ability to reduce the negative effects on the environment of customers’ trust in a brand's product or service are defined (Zhou et al., 2021). Environment‐friendly products and green brand awareness motivate customers who value the environment in their purchasing decisions. It is the responsibility of marketers to provide green product information and environment‐friendly labels by using content through green messages to educate consumers about their green brands (Chaihanchanchai & Anantachart, 2023). In addition, a green brand is a perception and association in the minds of consumers that the brand is connected to and interested in the environment (Ahmad et al., 2022). The elements of a green brand are divided into green brand image (Tan et al., 2022), green satisfaction, and green trust (Ha et al., 2022).

Some studies have empirically demonstrated the relationship between brand awareness and marketing activities. Borah et al. (2021) revealed that there is a significant relationship between the level of green brand knowledge of consumers and the marketing activities of companies. Alamsyah et al. (2018) concluded that product promotion ads organized with the theme of green marketing enable consumers who are sensitive to environmental products to exhibit purchasing behavior. Green brand awareness can be transformed into purchasing behavior through green marketing activities (Pancić et al., 2023). On the other hand, Nguyen‐Viet (2022a) found that the elements of a green marketing mix effectively ensure green customer‐based brand equity. Another study revealed that eco‐label and green content advertising activities affect green purchasing behavior. In this process, consumers’ green brand awareness has been proposed as a prerequisite (Nguyen‐Viet, 2022b). In light of this information, hypotheses of the conceptual model were formed as follows:

**H_12_**. Consumers’ green brand awareness is significantly related to green marketing activities.

Studies have proven the relationship between green brand awareness and planned behavior (Amoako et al., 2020; Asif et al., 2023; Wu et al., 2022). Based on this, the following hypotheses were established.

**H_4_**. Consumers’ green brand awareness significantly affects their ATT.
**H_5_**. Consumers’ green brand awareness significantly affects their SBNs.
**H_6_**. Consumers’ green brand awareness significantly affects their perceived behavior control.

Consumers’ green brand awareness directs their purchasing behavior (Rahmadhani & Widodo, 2023; Siyal et al., 2021; Zhou et al., 2021). Thus, the following hypothesis was proposed.

**H_11_**. Consumers’ green brand awareness significantly affects their purchase intentions.

### 2.3 Extended planned behavior theory in marketing

The TPB was proposed to improve the predictive power of the theory of reasoned action, which addresses the process of individuals’ ATTs turning into behavior (Ajzen, 1985). The theory of planned action assumes that human behavior is goal‐directed. Therefore, people need to do what to do to reach their goals. Constantly repetitive behaviors become routine and automatic. However, highly developed skills often no longer require the formulation of a conscious behavioral plan (Ajzen, 1991). Green purchasing intentions also require consumers to be knowledgeable about products and brands (Correia et al., 2023). Consumers are guided by factors such as internal motivations (Silvi & Padilla, 2021), formal education (Malik et al., 2019), and marketing activities (Mehraj et al., 2023; Tan et al., 2022), enabling them to gain green awareness.

The extended TPB has also been used by marketing researchers to predict customer behavior. Hameed et al. (2019) proved that the subdimensions of the theory of planned action have a mediating role in predicting environmentally conscious consumer behavior. Consumer behavior is the result of a conscious process. This process is affected by many variables. Although consumers’ environmental awareness affects their purchasing behavior, it also initiates the process of planned behavior. Planned behavior mediates this process (Wei et al., 2021). Consumers can be affected by many stimuli, have environmental concerns, and shape their future behavior. This includes purchasing behavior. In this respect, environmental concern is an important predictor of purchasing behavior. Planned behavior has a mediating effect that increases the effect of environmental concern in this process (Kumar et al., 2022). ATTs toward green products, SBNs toward green products, and green behavioral control affect green purchasing behavior and push consumers to purchase green products (Qazi et al., 2023). Consumers’ moral norms also affect their purchasing intentions. In this process, the dimensions of planned behavior strengthen the relationship between variables by mediating the interaction (Liu et al., 2020).

Based on the previously discovered empirical results, the following hypotheses were created:

**H_7_**. The ATT toward purchasing green products is positively associated with green purchase intentions.
**H_8_**. SBNs regarding buying green products are positively associated with green purchase intentions.
**H_9_**. Perceived behavioral control norms regarding purchasing green products are positively related to green purchase intentions.

### 2.4 Consumer purchase intention

Purchase intention refers to the intention of consumers to consciously purchase brand products (Irfany et al., 2023). Behavioral intentions, on the other hand, can be predicted from ATTs (Liu et al., 2020). Consumer behavior involves studying the various procedures that individuals, groups, or organizations adopt to select and dispose of products, services, or experiences to meet their needs, as well as the impact of these procedures on society (Mehraj et al., 2023). It is difficult to influence behavior without affecting ATTs and values. However, McGuire (1989) noted that claims and ATTs may not always translate into real behavior. Purchase intention is closely related to trust. Studies have confirmed that the trust factor significantly affects the purchasing intentions of customers (Tan et al., 2022; Wang, Zaman et al., 2022). On the other hand, advertisements play a key role in creating the expected results related to trust (Kwon et al., 2021). Advertising trust can reduce the perceived complexity and perceived risk in consumers’ purchase decision‐making process and increase the perceived certainty of expected results. Thus, it can positively influence purchase intentions (Sun et al., 2021). After consumers associate a brand with them, they will have a strong interest in the brand, and even brand loyalty (Panda et al., 2020) and brand commitment (Singh & Kunja, 2023) will develop. If consumers believe that a brand product can provide them with sentiment value or practical value, they likely have a strong intention to purchase a brand product to reap the benefits of that brand. Chand and Fei (2021) showed that consumers’ connections with a brand encourage purchasing intentions through their studies.

**FIGURE 1:**
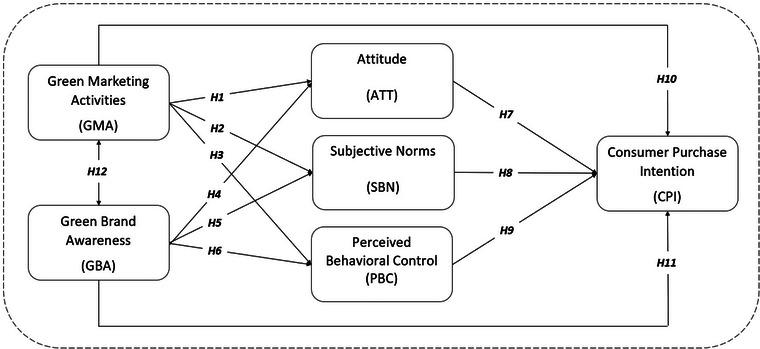
Conceptual model of the research. *Source*: Authors.

The conceptual model of the research, along with its hypotheses, is shown in Figure 1.

## 3 METHODOLOGY

### 3.1 The purpose of the study, the universe, and its sample

This study aims to structurally analyze the relationships among consumers’ green brand awareness levels, purchase intentions, and the green marketing activities of companies. In this context, university students born in the year 1995 and later, who are representatives of GEN Z, were determined as the universe of the research. Seven universities from Türkiye's Mediterranean Region, Southeastern Anatolia Region, Black Sea Region, and Central Anatolia regions were included in the research. The questionnaire forms created online were electronically distributed to the students. A random sampling method was used. A total of 638 questionnaires were returned. The questionnaires of 48 participants born before the year 1995 were not included in the study. Thus, the analyses carried out within the scope of the research were carried out on the remaining 590 survey data points.

### 3.2 Measurement tools used in the study

The primary data needed within the scope of the research were collected through online survey forms. The research scale used a 5‐point Likert‐type scale (1: I totally disagree…5: I totally agree). Four sections are included in the questionnaire. Demographic questions aimed at determining the characteristic features of the participants were placed in the first section. In the second part, to measure the green brand awareness of consumers, we conceptualized this measure by Keller (1993), and the validity and reliability of the study were provided by Yoo and Donthu (2001). A two‐dimensional scale was used. The five items included in the scale were adapted for assessing green brand awareness and were included in the questionnaire. In the third section, there are three questions used by Agostini et al. (2021) to measure the purchasing intentions of consumers. In the fourth section, the Green Marketing Activities Scale, which was coded in 15 articles by Sharma (2021) and collected under five themes, was included.

### 3.3 Findings

#### 3.3.1 Reliability of scales

In Table 1, the reliability coefficient numbers of the scales used in the research are presented. An examination of the data revealed that the reliability levels of the scales met the *α* ≥ .70 condition. Uyanah and Nsikhe (2023) defined the range of .61 < *α* < .80 as moderately reliable and the range of .81 < *α* < 1.00 as highly reliable for the scales. Accordingly, the green brand awareness (GBA) (*α* = .790) and consumer purchase intention (CPI) (*α* = .773) scales have moderate levels of reliability. The green marketing activities (GMA) (*α* = .819) and extended planned behavior (*α* = .734) scales are highly reliable.

**TABLE 1:** Results of reliability analysis.

Scale	Sizes	Item number	*α*	Source
Green brand awareness (GBA)	Remembering (RM)	3	.785	Keller (1993)
	Recognition (RC)	2	.792	
Consumer purchase intention (CPI)	Purchase intention (PI)	3	.773	Agostini et al. (2021)
Green marketing activities (GMA)	Product innovation and segmentation (PIS)	3	.824	Sharma (2021)
	Green promotion (GPO)	3	.842	
	Green branding (GRB)	3	.797	
	Green supply chain management (GSM)	3	.810	
	Green pricing (GPR)	3	.801	
Extended planned behavior (EPB)	Attitude (ATT)	3	.726	Liu et al. (2020)
	Subjective norms (SBNs)	4	.748	
	Perceived behavioral control (PBC)	3	.735	

*Source*: Authors.

#### 3.3.2 Demographic findings

The demographic findings of the participants were categorized through frequency analysis. According to these findings, more than half of the participating students were women (54.6%). A total of 9.2% of the participants were in the first grade, 20.3% were in the second grade, 32.9% were in the third grade, and 18.3% were in the fourth grade. The proportion of students studying in the field of science or health sciences was 50.3%. However, 29.7% of those studying in the field of social sciences were involved, and 20% were studying in the field of educational sciences. The remaining 19.3% of the participants had continued their undergraduate education. The vast majority of the participating students were single (89.2%). Overall, 60.2% of the participants had a monthly income of 2000 TL or less. A total of 11.5% of the participating students were 17 years old, 18.1% were 18 years old, 20.8% were 19, 15.8% were 20, 14.4% were 85, and 19.4% were 22. More than half of the students (52.3%) lived in dormitories (14.2% in private dormitories, 38.1% in state dormitories). Although the proportion of students who stay with their family is 5.6%, the proportion of those who stay in an apartment on their own is 14.7%, the proportion of those who stay in an apartment with friends is 11%, and the proportion of those who stay in apartment‐hotels is 16.3%. Sixty‐two percent of the participating students rate shopping on the Internet as safe. Thirty‐eight percent did not consider online shopping to be safe. Although 31% of the students shop online very often, 61% rarely use this channel. A small percentage (8%) of respondents did not prefer online shopping. The proportion of students who followed news about the environment was 60.5% (Table 2).

**TABLE 2:** Demographic information of the participants.

Gender	*n*	%	Marital status	*n*	%
Woman	322	54.6	The married	64	10.8
Boy	2684	45.4	single	526	89.2
Total	**590**	**100**	**Total**	**590**	**100**
Class	***n***	**%**	**Monthly income**	***n***	**%**
Best	54	9.2	Government scholarship/loan only	86	14.6
Second‐rate	120	20.3	851–1000 TL	103	17.5
Third grade	194	32.9	1001 L–2000 TL	166	28.1
Fourth grade	108	18.3	2001 L–3000 TL	114	19.3
Graduate	114	19.3	3001 TL and above	121	20.5
Total	**590**	**100**	**Total**	**590**	**100**
Age	***n***	**%**	**Where you are staying**	***n***	**%**
17	68	11.5	Next to his family	33	5.6
18	107	18.1	An apartment on your own	87	14.7
19	123	20.8	Apartment with friends	65	11
20	93	15.8	Aparthotel	96	16.3
21	85	14.4	Private dormitory	84	14.2
22	114	19.4	State dormitory	225	38.1
Total	**590**	**100**	**Total**	**590**	**100**
Internet shopping security	***n***	**%**	**Tracking news about the environment**	***n***	**%**
It is safer	366	62	I am following	357	60.5
it is not safe	224	38	I do not follow	233	39.5
Total	**590**	**100**	**Total**	**590**	**100**
Your area	***n***	**%**	**Frequency of online shopping**	***n***	**%**
**Science or health sciences**	296	50.3	Very often	183	31
**Social sciences**	175	29.7	Rarely	360	61
**Educational sciences**	118	20′	I'm not doing	47	8
Total	**590**	**100**	**Total**	**590**	**100**

*Source*: Authors.

#### 3.3.3 Arithmetic averages of factor structures, standard deviations, and correlations

The relationships between the factor structures constituting the scales used in the research were calculated by correlation decision analysis. The arithmetic averages and standard deviation coefficients were determined via frequency analysis. Accordingly, the two factors with the highest relationships were recognition (RC) and RM (*r* = .789; *p* < .001). There is a well‐directed and significant relationship between the level of RM green brands and the level of RC of green brands by GEN Z consumers. Another strong relationship was found between green promotion (GPO) and green branding (GRB) (*r* = .705; *p* < .001). There is a well‐directed and significant relationship between GEN Z consumers’ perceptions of GPO and GRB activities. The green pricing (GPR) was the variable with the lowest correlation with the other variables (RM for *r* = .208; *p* < .001; RC for *r* = .157; *p* < .001; purchase intention (PI) for *r* = .143; *p* < .001; product innovation and segmentation for *r* = .119; *p* < .001; GPO for *r* = .206; *p* < .001; GRB for *r* = .231; *p* < .001; ATT for *r* = .303; *p* < .001; SBN for *r* = .259; *p* < .001; and perceived behavioral control (PBC) for *r* = .463; *p* < .001). Considering that the arithmetic average of the green supply chain management variable is 3.08, it is concluded that the participant group has problems perceiving the activities of companies related to the green supply chain and does not have a clear idea about this phenomenon (Table 3).

**TABLE 3:** Arithmetic averages, standard deviations, and correlations.

	$\bar{**X}$	S.S.	1	2	3	4.	5	6	7	8	9	10	11
1. RM	4.01	0.584	1										
2. RC	3.97	0.478	0.789*	1									
3. PI	3.74	0.429	0.684*	0.671*	1								
4. PIS	3.26	0.590	0.245*	0.212*	0.201*	1							
5. GPO	3.83	0.367	0.653*	0.598*	0.625*	0.224*	1						
6. GRB	3.75	0.445	0.691*	0.637*	0.541*	0.326*	0.705*	1					
7. GSM	3.08	0.388	0.208*	0.157*	0.143*	0.119*	0.206*	0.231*	1				
8. GPR	3.62	0.411	0.324*	0.459*	−0.425*	0.217*	0.544*	0.438*	0.340*	1			
9. ATT	4.03	0.217	0.470*	486*	0.598*	0.278*	0.384*	0.477*	0.242*	0.303*	1		
10. SBN	3.57	0.569	0.392*	0.405*	0.468*	0.447*	0.360*	0.299*	0.293*	0.259*	0.388*	1	
11. PBC	3.79	0.431	0.387*	0.340*	0.433*	0.393*	0.409*	0.374*	0.227*	0.463*	0.271*	0.547*	1

$\bar{X}$: Arithmetic mean; P.Q: Standard deviation

*Significant compared to .001

Abbreviations: ATT, attitude; GPO, green promotion; GPR, green pricing; GRB, green branding; GSM, green supply chain management; PBC, perceived behavioral control; PI, purchase intention; PIS, product innovation and segmentation; RC, recognition; RM, remembering; SBN, subjective norms.

*Source*: Authors.

#### 3.3.4 Structural validity of the scales

The structural validity of the scales used in the research was tested by CFA. The results obtained are presented in Table 4. The confirmatory model formed according to these criteria showed a good fit (GFI: 0.91; AGFI: 0.88; CFI: 0.90; NFI: 0.92; RMSEA: 0.049; *x^2^/*df: 3.27; *p *≤ .05). For all factor sizes, the conditions of AVE ≥ .50, CR ≥ .70, and √AVE ≥ *r* were met. Thus, the assumptions of unity and dissociation validity were fulfilled for the factor structures tested (Serafini et al., 2016). The provision of the condition *t* ≥ 1.96 for all *t* values shows that the dimensions are significant in the ways created to measure the power of representing the scale (Baltaci, 2021) (Table 3).

**TABLE 4:** Confirmatory factor analysis (CFA) results and compliance indices.

Scale	Factor	*t*	AVE	CR	√AVE	Fit index
GBA	RM	19.75	.678	.798	.780	GFI: 0.91 AGFI: 0.88 CFI: 0.90 NFI: 0.92 RMSEA: 0.049 *x^2^*: 138.44 df: 42.29 *p*: .000
	RC	17.86	.684	.805	.827	
CPI	PI	18.37	.667	.782	.817	
GMA	PIS	14.20	.649	.754	.806	
	GPO	12.64	.676	.793	.822	
	GRB	13.42	.692	.817	.832	
	GSM	9.86	.579	.714	.761	
	GPR	15.63	.658	.760	.811	
EPB	ATT	14.29	.612	.778	.824	
	SBN	11.83	.630	.769	.816	
	PBC	13.74	.638	.783	.847	

Abbreviations: ATT, attitude; GPO, green promotion; GPR, green pricing; GRB, green branding; GSM, green supply chain management; PBC, perceived behavioral control; PI, purchase intention; PIS, product innovation and segmentation; RC, recognition; RM, remembering; SBN, subjective norms.

*Source*: Authors.

#### 3.3.5 Hypothesis testing through structural equation modeling

**FIGURE 2:**
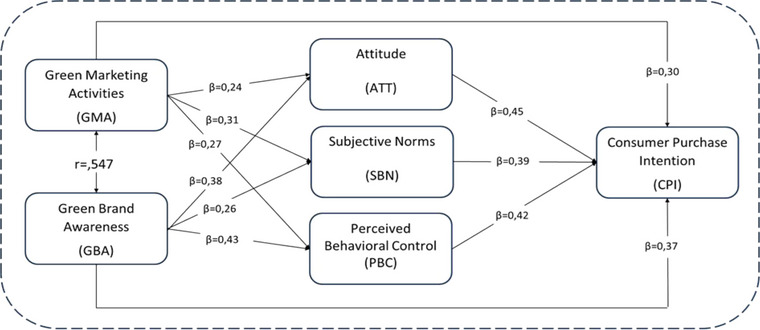
Structural equation modeling (SEM) for research hypotheses. *Source*: Authors.

SEM, which was created to test the research hypotheses, was tested through AMOS24. The obtained SEM output is presented in Figure 2. The fit indices show that the research model has a good fit and is structurally confirmed (*x^2^/*df: 3.27; GFI: 0.91; RMSEA: 0.049; *p* ≤ .05). The acceptance states of the hypotheses tested through the structural model are given in Table 5.

**TABLE 5:** Hypothesis testing results.

Hypotheses				*β*	S.H.	*t*	*p*	Result
H_1_	GMA		ATT	.245	.054	24.325	.002	Accept
H_2_	GMA		SBN	.314	.042	19.841	.004	Accept
H_3_	GMA		PBC	.273	.031	24.662	.003	Accept
H_4_	GBA		ATT	.381	.028	27.256	.000	Accept
H_5_	GBA		SBN	.262	.049	16.774	.008	Accept
H_6_	GBA		PBC	.433	.037	18.541	.006	Accept
H_7_	ATT		CPI	.451	.044	22.698	.004	Accept
H_8_	SBN		CPI	.390	.067	14.186	.003	Accept
H_9_	PBC		CPI	.422	.058	25.495	.001	Accept
H_10_	GMA		CPI	.335	.043	19.061	.001	Accept
H_11_	GBA		CPI	.371	.036	23.270	.002	Accept

S.H = Standard error

Abbreviations: ATT, attitude; GPO, green promotion; GPR, green pricing; GRB, green branding; GSM, green supply chain management; PBC, perceived behavioral control; PI, purchase intention; PIS, product innovation and segmentation; RC, recognition; RM, remembering; SBN, subjective norms.

*Source*: Authors.

When the data in Table 5 are examined, GMA has a statistically positive and significant effect on ATT (*β* = .245; *t* ≥ 1.96; *p* ≤ .05), SBN (*β* = .314; *t* ≥ 1.96; *p* ≤ .05), and PBC (*β* = .273; *t* ≥ 1.96; *p* ≤ .05). Thus, H_1_, H_2_, and H_3_ are accepted. GBA had a statistically positive and significant effect on ATT (*β* = .381; *t* ≥ 1.96; *p* ≤ .05), SBN (*β* = .262; *t* ≥ 1.96; *p* ≤ .05), and PBC (*β* = .433; *t* ≥ 1.96; *p *≤ .05). Thus, H_4_, H_5_, and H_6_ were accepted. ATT had a statistically positive and significant effect on the CPI (*β* = .351; *t* ≥ 1.96; *p* ≤ .05). Thus, H_7_ is accepted. SBN had a statistically significant positive effect on the CPI (*β* = .390; *t* ≥ 1.96; *p* ≤ .05). Thus, H_8_ is accepted. PBC had a statistically positive and significant effect on the CPI (*β* = .422; *t* ≥ 1.96; *p* ≤ .05). Thus, H_9_ is accepted. GMA had a statistically positive and significant effect on the CPI (*β* = .335; *t *≥ 1.96; *p* ≤ .05). Thus, H_10_ is accepted. GBA had a statistically positive and significant effect on the CPI (*β* = .371; *t* ≥ 1.96; *p* ≤ .05). Thus, H_11_ is accepted.

## 4 DISCUSSION

In this study, the relationships between GEN Z consumers’ green brand awareness, purchase intentions, and perceptions of green marketing activities were analyzed in a structural model within the scope of the TPB. Many studies have revealed that consumers’ brand perceptions affect their purchasing intentions (Febriyantoro, 2020; Pratama et al., 2023; Tan et al., 2021). Similar results were reached in this study, which revealed that consumers’ purchasing behaviors are governed by their green brand awareness. Andreani et al. (2021) revealed that GEN Z consumers’ green brand awareness directs their purchasing behavior. According to Barrón et al. (2022), Gen Z individuals exhibit more sensitive behavioral patterns in terms of environmental issues. The findings obtained in this study revealed that the level of green brand awareness of Gen Z is high, which supports the findings of the literature.

This study showed that consumers’ green brand awareness is important. This finding is also consistent with the literature. Asif et al. (2023) and Zhou et al. (2021) concluded that the level of green knowledge is a prerequisite for purchasing behavior. Thus, the findings of the study were theoretically supported.

The results obtained in the present study showed that there is a positive interaction effect between GEN Z consumers’ green brand awareness and their perceptions of green marketing activities. Pancić et al. (2023) showed that marketing activities carried out by companies are an important tool for creating brand awareness. Kusumah and Lee (2020) demonstrated that consumers’ green brand awareness can be managed through green brand marketing activities. Agostini et al. (2021) found that the perceived risk and perceived value of consumers have an impact on purchasing behavior. For this reason, they stated that brand awareness should increase and consumers’ risk perceptions should decrease. The way to do this is to increase the sellers’ reputation through marketing activities. Alamsyah et al. (2018) concluded that consumers’ green awareness can be guided by green marketing activities. In addition, a green brand image affects green awareness. For this reason, advertising and marketing activities are important for supporting green awareness among consumers (Zhou et al., 2021).

In some studies, it has been determined that green marketing regulates the relationships between customer value and ATTs toward green products and green purchasing intentions (Liao et al., 2020). The study concluded that consumers’ perceptions of green marketing activities affect their purchasing intentions. Borah et al. (2021) suggested that a new product activity is directly related to Green Sunday orientation, green innovation ability, green knowledge acquisition, and green brand positioning. Accordingly, the consideration and successful implementation of these variables in green marketing activities will also positively affect consumer purchasing tendencies. Jaiswal et al. (2021) revealed that stimulants are important in triggering purchasing behavior in green marketing activities. SEM confirmed that eco‐labels, eco‐brands, and green advertisements are the leading stimulants among these stimulants.

Nguyen‐Viet (2022a) noted that all elements of the marketing mix should be reconsidered as a green concept. Green products, GPR, green places, and GPO activities will create consumers’ green trust and green loyalty. Thus, purchasing ATTs will be converted into behavior and reinforced. In another study, Nguyen‐Viet (2022b) found that eco‐labels and green advertisements used within the scope of marketing activities positively affect purchasing behavior. Mehraj and Qureshi (2022), on the other hand, focused on positioning within marketing activities. The results obtained have shown that green positioning strategies that integrate the functional qualities of products and services with emotional benefits have a positive effect on consumer ATTs at a high level.

Vasan (2023) revealed that social media posts and Internet clicks support purchasing behavior. The rate at which GEN Z spends time on the Internet is quite high. Prakash Yadav and Rai (2017) revealed that GEN Z spends much time on social media and the Internet, triggering behaviors that also have social outcomes. In this study, almost all of the participants made purchases via the Internet, and the vast majority of them described it as safe. This finding confirms that online environments are an important and effective tool for carrying out activities to trigger green buying behavior in GEN Z and increase green brand awareness. Andreani et al. (2021) strengthened this knowledge by concluding that brand awareness supported by social media phenomena positively affects purchasing behavior. GEN Z consumers’ green ATTs, green social norms, green purchasing intentions, and green purchasing behaviors interact. GEN Z consumers are more interested in environmental issues (Qazi et al., 2023).

This situation reflects a planned process. Green marketing activities increase consumers’ awareness and induce green purchasing intentions and green purchasing behavior. The findings obtained in our study also support this. The GEN Z participants’ green brand awareness and perception of green marketing activities interact. Thus, they also affect ATT, SBN, and PBC, which are subcomponents of the TPB. Amoako et al. (2020) showed that the theory of planned action can be used to predict young peoples’ green purchasing processes. Accordingly, the level of green knowledge has a positive effect on ATTs and norms.

Based on the TPB, ATT, SBN, and PBC are considered mandatory indicators of green product purchase intention (Kamalanon et al., 2022). The results we obtained in our study confirmed this finding. Three factors based on the TPB significantly affect purchase intentions (Sousa et al., 2022). Some studies have also emphasized that SBNs do not have a significant effect on purchase intentions. This finding shows that sample variation may affect ATTs through the cultural perspective. There are similar results in the literature. Pan et al. (2022) found that ATT, SBN, and PBC positively and significantly affect green purchase intentions. Wang et al. (2022) noted the positive impact of consumer ATTs, SBN, behavioral controls, and green purchasing behavior. Shukla (2019) proved that the theory of planned action can be used to predict consumers’ green purchasing behavior. The results of Almrafee and Akaileh (2023) showed that ATTs, SBN (social influence), and PBC significantly influence customers’ purchase intention to adopt green‐friendly products. Wongsaichia et al. (2022) results proved that SBN, behavioral control, and ATTs mediate the relationship between environmental concern and purchase intentions. Patwary et al. (2022) concluded that environmental advertisements affect the behavioral intention to purchase, and that ATT, SBN, and PBC mediate this interaction. Thus, the mediation effect obtained in our study was supported by the literature.

## 5 CONCLUSION

This study proved that consumers’ purchasing intentions can be guided by green marketing activities and green brand awareness. Informing consumers through green‐themed marketing activities increases green brand awareness. However, it encourages them to be planned during the process. Consumers who become aware of green brands through advertising and marketing channels display a planned behavior in their purchasing processes and turn to green products. Green brand awareness and green marketing activities already positively affect the purchasing process. The planned behavior of consumers with increased consciousness and awareness levels catalyzes the purchasing intention positively. In other words, it strengthens the effect of green brand awareness on purchasing intention through green marketing activities.

The mediating role of planned behavior in the working model is considered important, because the effect of direct independent variables may be insufficient to explain a model. In addition, the variables that provide the mediation effect are considered small details that provide important competitive power for the parties. The three dimensions discussed specifically in the TPB strengthen the effect of green marketing activities and green brand awareness on purchasing intention to a similar extent. It proves the fact that each dimension of planned behavior is of equal importance and that none should be ignored in the calculations. It should not be ignored that the sample also has an impact on the verification of research hypotheses and the experience of expected results. Many studies on GEN Z have shown that individuals of this generation learn quickly and can adapt easily. In other words, their readiness levels are quite high. The significant correlation between green marketing activities and green brand awareness also supports this claim. Although the marketing tools to be created for GEN Z consumers will push them into a planned behavior process, their ability to exhibit the behavior expected by businesses depends on the creation of a good brand image perception. The results obtained in the study showed that GEN Z consumers are sensitive to nature‐friendly and green‐image products. Planned behavior dimensions that induce purchasing behavior demonstrate this. Namely, strengthening the green brand image among GEN Z consumers through green marketing activities creates a strong ATT and activates the planned purchasing process. Similarly, GEN Z consumers tend to make planned purchases through stimulation of their PBC levels. SBNs are not as effective as others in this process. These results revealed the purchasing process cycle of GEN Z consumers around an empirical model. The paper expanded the strategies that businesses should adopt with the TPB and offered clues to ensure competitiveness.

## 6 CONTRIBUTION AND FUTURE DIRECTIONS

### 6.1 Theoretical contribution

This study focused on the impact of consumers’ perceived green marketing activities and green brand awareness on purchase intentions. This interaction process was expanded by the TPB. The conceptual model supported by the literature was empirically validated with a quantitative research design. Thus, a structural model with proven validity and reliability was presented in the literature. The study discussion of the green purchasing process within the framework of multidimensional planned action theory offered an up‐to‐date perspective on the literature.

The results obtained in the present study revealed that online tools will be effective in green brand awareness studies and green brand marketing activities related to GEN Z. However, it has been proven that GEN Z is also a classification that should be considered in the demographic segmentation process. In addition, the fact that GEN Z consumers with high perceptions of green brands can represent the green class proposed by Qazi et al. (2023) has been clearly shown in the study. Finisterra do Paco and Raposo (2009) stated that the market can be divided according to certain criteria; two studies have been conducted in which age group and green emphasis can be used as criteria. Park and Lee (2014) collected data on consumers in four classes according to their ATTs toward green brands. The fact that the group with the highest level of corporate social responsibility, which has green awareness and a pronounced sense of environmentalism and is in the age range of 18–44, includes GEN Z has attracted attention as an important detail.

Barrón et al. (2022) identified GEN Z as an important generation for preventing climate change. Therefore, they emphasized that it is important for them to adopt a behavioral pattern that has a pro‐environmental and green image. The findings obtained in this study show that GEN Z has a high perception of green brands. It is important to catalyze this perception through green marketing activities to transform it into behavior.

The results obtained showed that the participants’ perceptions about the supply chain are extremely low, and they remain undecided about it. The fact that supply chain management is not in the direct consumers’ field of interest is the main factor that leads to this result. However, many companies transfer the entire process from production to consumption to their consumers through tools such as square codes, geographical indications, and advertisements. This situation provides an important advantage for companies that want to achieve competitiveness and want to reach their consumers with the slogan “transparency” (Khan et al., 2023).

### 6.2 Practical contribution

The study has revealed important results both for businesses and for planners and policymakers. This study, which response to the creation of brand perception and awareness among consumers, the structuring of marketing activities, and the transformation of purchasing intentions into behaviors among consumers with empirical findings, is a guide for subsequent studies. GEN Z has high green brand awareness. Additionally, green advertising and brand awareness are positively related. Emphasizing the environment in advertisements will enable GEN Z consumers to exhibit planned behavior and induce purchasing behavior.

### 6.3 Limitations and future directions

All studies have limitations, and this is no exception. The primary data of this study were collected in Türkiye. Therefore, it needs to be repeated in other samples to be generalized. This study discussed advertising activities and brand awareness within the scope of GEN Z. Comparing the results with studies on Generations Y and X will contribute to the marketing literature. Additionally, including demographic variables such as age, sex, monthly income, and education level in the model will help achieve different results. The study focused on GEN Z studying at university. The research used the TPB as a mediating variable. Addressing the regulatory effect in future studies will contribute to the expansion of the literature. Green products were centered in the study. It would be interesting if researchers test the tested model in nongreen products and reveal the mediating effect of planned behavior. In addition, adding digitalization, digital skills, and technological readiness variables to the model will provide more effective results for GEN Z.

## AUTHOR CONTRIBUTIONS

**Duygu Baltacı**: Conceptualization; data curation; investigation; resources; software; writing—original draft. **Yakup Durmaz**: Formal analysis; funding acquisition; project administration; supervision; writing—review and editing. **Furkan Baltacı**: Methodology; resources; software; writing—original draft; writing—review and editing.

## CONFLICT OF INTEREST STATEMENT

The authors declare that they have no known conflicts of interest or personal relationships that could have appeared to influence the work reported in this paper.

## FUNDING INFORMATION

No funds were received from any institution.

### PEER REVIEW

The peer review history for this paper is available at https://publons.com/publon/10.1002/brb3.3584.

## Data Availability

Data will be made available on request.

## References

[brb33584-bib-0001] Agostini, L. , Bigliardi, B. , Filippelli, S. , & Galati, F. (2021). Seller reputation, distribution, and intention to purchase refurbished products. Journal of Cleaner Production, 316, 128296. 10.1016/j.jclepro.2021.128296

[brb33584-bib-0002] Ahmad, F. , Rosli, N. T. , & Quoquab, F. (2022). Environmental quality awareness, green trust, green self‐efficacy and environmental attitude in influencing green purchase behavior. International Journal of Ethics and Systems, 38(1), 68–90. 10.1108/IJOES-05-2020-0072

[brb33584-bib-0003] Ajzen, I. (1985). From intentions to actions: A theory of planned behavior. In Action control: From cognition to behavior (pp. 11–39). Springer Berlin Heidelberg. 10.1007/978-3-642-69746-3_2

[brb33584-bib-0004] Ajzen, I. (1991). The theory of planned behavior. Organizational Behavior and Human Decision Processes, 50(2), 179–211. 10.1016/0749-5978(91)90020-T

[brb33584-bib-0005] Ajzen, I. , Fishbein, M. , Lohmann, S. , & Albarracín, D. (2018). The influence of attitudes on behavior. In The handbook of attitudes, Vol. 1: Basic principles (pp. 197–255). Routledge.

[brb33584-bib-0006] Alaloul, W. S. , Liew, M. S. , Zawawi, N. A. W. A. , & Kennedy, I. B. (2020). Industrial revolution 4.0 in the construction industry: Challenges and opportunities for stakeholders. Ain Shams Engineering Journal, 11(1), 225–230. 10.1016/j.asej.2019.08.010

[brb33584-bib-0007] Alamsyah, D. P. , & Febriani, R. (2020). Green customer behaviour: Impact of green brand awareness to green trust. Journal of Physics: Conference Series, 1477(7), 072022. 10.1088/1742-6596/1477/7/072022

[brb33584-bib-0008] Alamsyah, D. P. , Suhartini, T. , Rahayu, Y. , Setyawati, I. , & Hariyanto, O. I. (2018). Green advertising, green brand image, and green awareness for environmental products. In IOP Conference Series: Materials Science and Engineering, 434(1), 1–7. 10.1088/1757-899X/434/1/012160

[brb33584-bib-0009] Almrafee, M. , & Akaileh, M. (2023). Customers' purchase intention of renewable energy in Jordan: The case of solar panel systems using an extended theory of planned behavior (TPB). International Journal of EnergySector Management, 18(5), 457–473. 10.1108/IJESM-01-2023-0002

[brb33584-bib-0010] Alsharif, A. H. , & Alharbi, I. B. (2022). Scientometric analysis of scientific literature on neuromarketing tools in advertising. Baltic Journal of Economic Studies, 8(5), 1–12. 10.30525/2256-0742/2022-8-5-1-12

[brb33584-bib-0011] Alsharif, A. H. , Salleh, N. Z. M. , Alrawad, M. , & Lutfi, A. (2023). Exploring global trends and future directions in advertising research: A focus on consumer behavior. Current Psychology, 43, 1–24. 10.1007/s12144-023-04812-w PMC10239056 37359681

[brb33584-bib-0012] Alsharif, A. H. , Salleh, N. Z. M. , Al‐Zahrani, S. A. , & Khraiwish, A. (2022). Consumer behavior to be considered in advertising: A systematic analysis and future agenda. Behavioral Sciences, 12(12), 472. 10.3390/bs12120472 36546955 PMC9774318

[brb33584-bib-0013] Alsharif, A. H. , Salleh, N. Z. M. , Hashem, E. A. R. , Khraiwish, A. , Putit, L. , & Arif, L. S. M. (2023). Exploring factors influencing neuromarketing implementation in Malaysian Universities: Barriers and enablers. Sustainability, 15(5), 4603. 10.3390/su15054603

[brb33584-bib-0014] Amoako, G. K. , Dzogbenuku, R. K. , & Abubakari, A. (2020). Do green knowledge and attitude influence the youth's green purchasing? Theory of planned behavior. International Journal of Productivity and Performance Management, 69(8), 1609–1626. 10.1108/IJPPM-12-2019-0595

[brb33584-bib-0015] Andreani, F. , Gunawan, L. , & Haryono, S. (2021). Social media influencer, brand awareness, and purchase decision among generation Z in Surabaya. JurnalManajemen Dan Kewirausahaan, 23(1), 18–26. 10.9744/jmk.23.1.18-26

[brb33584-bib-0016] Arya, V. , Sambyal, R. , Sharma, A. , & Dwivedi, Y. K. (2024). Brands are calling your AVATAR in metaverse—A study to explore XR‐based gamification marketing activities & consumer‐based brand equity in virtual world. Journal of Consumer Behaviour, 23(2), 556–585. 10.1002/cb.2214

[brb33584-bib-0017] Asif, M. H. , Zhongfu, T. , Irfan, M. , & Işık, C. (2023). Do environmental knowledge and green trust matter for purchase intention of eco‐friendly home appliances? An application of extended theory of planned behavior. Environmental Science and Pollution Research, 30(13), 37762–37774. 10.1007/s11356-022-24899-1 36574131

[brb33584-bib-0111] Badi, S. , & Murtagh, N. (2019). Green supply chain management in construction: A systematic literature review and future research agenda. Journal of Cleaner Production, 223, 312–322. 10.1016/j.jclepro.2019.03.132

[brb33584-bib-0018] Baltaci, F. (2021). The relationship between the environmental attitude, behavioral role and support of the local resident in sustainable development of tourism: The example of Alanya. Journal of Economy Culture and Society, 63, 213–236. 10.26650/JECS2020-0067

[brb33584-bib-0019] Barrón, N. G. , Gruber, S. , & Huffman, G. (2022). Student engagement and environmental awareness: Gen Z and ecocomposition. Environmental Humanities, 14(1), 219–232. 10.1215/22011919-9481528

[brb33584-bib-0020] Bhardwaj, A. , Alam, T. , Sharma, V. , Alam, M. S. , Hamid, H. , & Deshwal, G. K. (2020). Lignocellulosic agricultural biomass as a biodegradable and eco‐friendly alternative for polymer‐based food packaging. Journal of Packaging Technology and Research, 4, 205–216. 10.1007/s41783-020-00089-7

[brb33584-bib-0021] Bi, C. , Jin, S. , & Li, S. (2023). Can green advertising increase consumer purchase intention of electric vehicles? An experimental study from China. Journal of Cleaner Production, 419, 138260. 10.1016/j.jclepro.2023.138260

[brb33584-bib-0022] Borah, P. S. , Dogbe, C. S. K. , Pomegbe, W. W. K. , Bamfo, B. A. , & Hornuvo, L. K. (2021). Green market orientation, green innovation capability, green knowledge acquisition and green brand positioning as determinants of new product success. European Journal of Innovation Management, 26(2), 364–385. 10.1108/EJIM-09-2020-0345

[brb33584-bib-0023] Chaihanchanchai, P. , & Anantachart, S. (2023). Encouraging green product purchase: Green value and environmental knowledge as moderators of attitude and behavior relationship. Business Strategy and the Environment, 32(1), 289–303. 10.1002/bse.3130

[brb33584-bib-0024] Chand, V. S. , & Fei, C. (2021). Self‐brand connection and intention to purchase a counterfeit luxury brand in emerging economies. Journal of Consumer Behaviour, 20(2), 399–411. 10.1002/cb.1871

[brb33584-bib-0025] Cheung, M. F. , & To, W. M. (2019). An extended model of value‐attitude behaviour to explain Chinese consumers' green purchase behavior. Journal of Retailing and Consumer Services, 50, 145–153. 10.1016/j.jretconser.2019.04.006

[brb33584-bib-0026] Correia, E. , Sousa, S. , Viseu, C. , & &Larguinho, M. (2023). Analyzing the influence of green marketing communication in consumers’ green purchase behavior. International Journal of Environmental Research and Public Health, 20(2), 1356. 10.3390/ijerph20021356 36674112 PMC9858907

[brb33584-bib-0028] Dai, J. , & Sheng, G. (2022). Advertising strategies and sustainable development: The effects of green advertising appeals and subjective busyness on green purchase intention. Business Strategy and the Environment, 31(7), 3421–3436. 10.1002/bse.3092?saml_referrer

[brb33584-bib-0029] Febriyantoro, M. T. (2020). Exploring YouTube marketing communication: Brand awareness, brand image and purchase intention in the millennial generation. Cogent Business & Management, 7(1), 1787733. 10.1080/23311975.2020.1787733

[brb33584-bib-0029a] Finisterra do Paço, A. M. , Barata Raposo, M. L. , & Filho, W. L. (2009). Identifying the green consumer: A segmentation study. Journal of Targeting, Measurement and Analysis for Marketing, 17, 17–25. 10.1057/jt.2008.28

[brb33584-bib-0030] Freeman, J. J. , & Nunez, K. M. (2020). Waste anaesthetic gases cost UNMH money and contribute to our institutional carbon footprint. Annual Quality Improvement & Patient Safety Symposium, The University Of New Mexico. https://digitalrepository.unm.edu/hsc_qips/35/

[brb33584-bib-0031] Gómez‐Rico, M. , Molina‐Collado, A. , Santos‐Vijande, M. L. , Molina‐Collado, M. V. , & Imhoff, B. (2023). The role of novel instruments of brand communication and brand image in building consumers’ brand preference and intention to visit wineries. Current Psychology, 42(15), 12711–12727. 10.1007/s12144-021-02656-w PMC8740867 35035183

[brb33584-bib-0032] Ha, M. T. , Ngan, V. T. K. , & Nguyen, P. N. (2022). Greenwash and green brand equity: The mediating role of green brand image, green satisfaction and green trust and the moderating role of information and knowledge. Business Ethics, the Environment & Responsibility, 31(4), 904–922. 10.1111/beer.12462

[brb33584-bib-0033] Hameed, I. , Waris, I. , & Amin ul Haq, M. (2019). Predicting eco‐conscious consumer behavior using theory of planned behavior in Pakistan. Environmental Science and Pollution Research, 26, 15535–15547. 10.1007/s11356-019-04967-9 30945077

[brb33584-bib-0034] Irfany, M. I. , Khairunnisa, Y. , & Tieman, M. (2023). Factors influencing Muslim generation Z consumers’ purchase intention of environmentally friendly halal cosmetic products. Journal of Islamic Marketing, 15(1), 221–243. 10.1108/JIMA-07-2022-0202

[brb33584-bib-0035] Jaiswal, D. , Singh, B. , Kant, R. , & Biswas, A. (2021). Toward green product consumption: Effect of green marketing stimuli and perceived environmental knowledge in Indian consumer market. Society and Business Review, 17(1), 45–65. 10.1108/SBR-05-2021-0081

[brb33584-bib-0036] Kamalanon, P. , Chen, J. S. , & Le, T. T. Y. (2022). “Why do we buy green products?” An extended theory of the planned behavior model for green product purchase behavior. Sustainability, 14(2), 689. 10.3390/su14020689

[brb33584-bib-0037] Keller, K. L. (1993). Conceptualizing, measuring, and managing customer‐based brand equity. Journal of Marketing, 57(1), 1–22. 10.1177/002224299305700101

[brb33584-bib-0038] Khan, I. , Hou, F. , & Le, H. P. (2021). The impact of natural resources, energy consumption, and population growth on environmental quality: Fresh evidence from the United States of America. Science of the Total Environment, 754, 142222. 10.1016/j.scitotenv.2020.142222 32920417

[brb33584-bib-0039] Khan, M. , Ajmal, M. M. , Jabeen, F. , Talwar, S. , & Dhir, A. (2023). Green supply chain management in manufacturing firms: A resource‐based viewpoint. Business Strategy and the Environment, 32(4), 1603–1618. 10.1002/bse.3207

[brb33584-bib-0040] Kimiagari, S. , & Malafe, N. S. A. (2021). The role of cognitive and affective responses in the relationship between internal and external stimuli on online impulse buying behavior. Journal of Retailing and Consumer Services, 61, 102567. 10.1016/j.jretconser.2021.102567

[brb33584-bib-0041] Kinnunen, J. , Saunila, M. , Ukko, J. , & Rantanen, H. (2022). Strategic sustainability in the construction industry: Impacts on sustainability performance and brand. Journal of Cleaner Production, 368, 133063. 10.1016/j.jclepro.2022.133063

[brb33584-bib-0042] Ko, E. , Costello, J. P. , & Taylor, C. R. (2019). What is a luxury brand? A new definition and review of the literature. Journal of Business Research, 99, 405–413. 10.1016/j.jbusres.2017.08.023

[brb33584-bib-0043] Kotler, P. , & Armstrong, G. (1995). Principles of marketing (7th ed.). Prentice Hall.

[brb33584-bib-0044] Kotler, P. , & Keller, K. (2006). Marketing management (12th ed.). Pearson/Prentice Hall.

[brb33584-bib-0045] Kumar, N. , Garg, P. , & Singh, S. (2022). Pro‐environmental purchase intention toward eco‐friendly apparel: Augmenting the theory of planned behavior with perceived consumer effectiveness and environmental concern. Journal of Global Fashion Marketing, 13(2), 134–150. 10.1080/20932685.2021.2016062

[brb33584-bib-0046] Kusumah, A. , & Lee, C. W. (2020). Do green marketing and green brand awareness influence customer satisfaction? An empirical study. Oradea Journal of Business and Economics, 5(2), 31–43. 10.47535/1991ojbe109

[brb33584-bib-0047] Kwon, J. H. , Jung, S. H. , Choi, H. J. , & Kim, J. (2021). Antecedent factors that affect restaurant brand trust and brand loyalty: Focusing on US and Korean consumers. Journal of Product & Brand Management, 30(7), 990–1015. 10.1108/JPBM-02-2020-2763

[brb33584-bib-0048] Liao, Y. K. , Wu, W. Y. , & Pham, T. T. (2020). Examining the moderating effects of green marketing and green psychological benefits on customers’ green attitude, value and purchase intention. Sustainability, 12(18), 7461. 10.3390/su12187461

[brb33584-bib-0049] Lin, C. C. , & Dong, C. M. (2023). Exploring Consumers' purchase intention on energy‐efficient home appliances: Integrating the theory of planned behavior, perceived value theory, and environmental awareness. Energies, 16(6), 2669. 10.3390/en16062669

[brb33584-bib-0050] Lin, H. , Gao, J. , & Tian, J. (2022). Impact of tourist‐to‐tourist interaction on responsible tourist behavior: Evidence from China. Journal of Destination Marketing & Management, 24, 100709. 10.1016/j.jdmm.2022.100709

[brb33584-bib-0051] Liu, M. T. , Liu, Y. , & Mo, Z. (2020). Moral norm is the key: An extension of the theory of planned behavior (TPB) on Chinese consumer's green purchase intention. Asia Pacific Journal of Marketing and Logistics, 32(8), 1823–1841. 10.1108/APJML-05-2019-0285

[brb33584-bib-0052] Malik, M. I. , Nawaz Mir, F. , Hussain, S. , Hyder, S. , Anwar, A. , Khan, Z. U. , & Waseem, M. (2019). Contradictory results on environmental concern while revisiting green purchase awareness and behavior. Asia Pacific Journal of Innovation and Entrepreneurship, 13(1), 17–28. 10.1108/APJIE-11-2018-0061

[brb33584-bib-0053] Mammadli, M. (2023). Factors driving sustainable consumption in Azerbaijan: Comparison of generation X, generation Y and generation Z. Sustainability, 15(20), 15159. 10.3390/su152015159

[brb33584-bib-0054] McDaniel, S. W. , & Kolari, J. W. (1987). Marketing strategy implications of the Miles and Snow strategic typology. Journal of Marketing, 51(4), 19–30. 10.1177/002224298705100403

[brb33584-bib-0055] McGuire, W. J. (1989). The structure of individual attitudes and attitude systems. In A. R. Pratkanis , S. J. Breckler , & A. G. Greenwald (Eds.), Attitude structure and function (pp. 37–69). Psychology Press.

[brb33584-bib-0056] Meadows, D. H. , Meadows, D. L. , Randers, J. , & Behrens, W. W. (1972). The limits to growth. Universe Books.

[brb33584-bib-0057] Mehraj, D. , & Qureshi, I. H. (2022). Does green brand positioning translate into green purchase intention? A mediation–moderation model. Business Strategy and the Environment, 31(7), 3166–3181. 10.1002/bse.3069

[brb33584-bib-0058] Mehraj, D. , Qureshi, I. H. , Singh, G. , Nazir, N. A. , Basheer, S. , & Nissa, V. U. (2023). Green marketing practices and green consumer behavior: Demographic differences among young consumers. Business Strategy & Development, 6(4), 571–585. 10.1002/bsd2.263

[brb33584-bib-0059] Miles, R. E. , Snow, C. C. , Meyer, A. D. , & Coleman, H. J. (1978). Organizational strategy, structure and process. The Academy of Management Review, 3(3), 546–562. 10.5465/amr.1978.4305755 10238389

[brb33584-bib-0060] Nekmahmud, M. , & Fekete‐Farkas, M. (2020). Why not green marketing? Determinates of consumers’ intention to green purchase decision in a new developing nation. Sustainability, 12(19), 7880. 10.3390/su12197880

[brb33584-bib-0061] Nguyen‐Viet, B. (2022a). The impact of green marketing mix elements on green customer based brand equity in an emerging market. Asia‐Pacific Journal of Business Administration, 15(1), 96–116. 10.1108/APJBA-08-2021-0398

[brb33584-bib-0062] Nguyen‐Viet, B. (2022b). Understanding the influence of eco‐label, and green advertising on green purchase intention: The mediating role of green brand equity. Journal of Food Products Marketing, 28(2), 87–103. 10.1080/10454446.2022.2043212

[brb33584-bib-0063] Obel, B. , & Gurkov, I. (2023). Using old theories to find novel solutions in organizational design of large established firms. International Journal of Organizational Analysis, 31(6), 2372–2382. 10.1108/IJOA-11-2021-3054

[brb33584-bib-0064] Ozkara, B. Y. , & Bagozzi, R. (2021). The use of event related potentials brain methods in the study of conscious and unconscious consumer decision making processes. Journal of Retailing and Consumer Services, 58, 102202. 10.1016/j.jretconser.2020.102202

[brb33584-bib-0065] Padilla‐Lozano, C. P. , & Collazzo, P. (2021). Corporate social responsibility, green innovation and competitiveness–causality in manufacturing. Competitiveness Review: An International Business Journal, 32(7), 21–39. 10.1108/CR-12-2020-0160

[brb33584-bib-0066] Pan, J. , Teng, Y. M. , Wu, K. S. , & Wen, T. C. (2022). Anticipating Z‐generation tourists’ green hotel visit intention utilizing an extended theory of planned behavior. Frontiers in Psychology, 13, 1008705. 10.3389/fpsyg.2022.1008705 36562051 PMC9764080

[brb33584-bib-0067] Pancić, M. , Serdarušić, H. , & Ćućić, D. (2023). Green marketing and repurchase intention: Stewardship of green advertisement, brand awareness, brand equity, green innovativeness, and brand innovativeness. Sustainability, 15(16), 12534. 10.3390/su151612534

[brb33584-bib-0068] Panda, T. K. , Kumar, A. , Jakhar, S. , Luthra, S. , Garza‐Reyes, J. A. , Kazancoglu, I. , & Nayak, S. S. (2020). Social and environmental sustainability model on consumers’ altruism, green purchase intention, green brand loyalty and evangelism. Journal of Cleaner Production, 243, 118575. 10.1016/j.jclepro.2019.118575

[brb33584-bib-0069] Park, J. S. , & Lee, J. (2014). Segmenting green consumers in the United States: Implications for green marketing. Journal of Promotion Management, 20(5), 571–589. 10.1080/10496491.2014.946202

[brb33584-bib-0070] Parnell, J. A. , & Wright, P. (1993). Generic strategy and performance: An empirical test of the Miles and Snow typology. British Journal of Management, 4(1), 29–36. 10.1111/j.1467-8551.1993.tb00159.x

[brb33584-bib-0071] Patel, N. , & Mehta, D. (2023). The asymmetry effect of industrialization, financial development and globalization on CO_2_ emissions in India. International Journal of Thermofluids, 20, 100397. 10.1016/j.ijft.2023.100397

[brb33584-bib-0072] Patwary, A. K. , Mohamed, M. , Rabiul, M. K. , Mehmood, W. , Ashraf, M. U. , & Adamu, A. A. (2022). Green purchasing behavior of international tourists in Malaysia using green marketing tools: Theory of planned behavior perspective. Nankai Business Review International, 13(2), 246–265. 10.1108/NBRI-06-2021-0044

[brb33584-bib-0110] Pan, J. , Teng, Y. M. , Wu, K. S. , & Wen, T. C. (2022). Anticipating Z‐generation tourists’ green hotel visit intention utilizing an extended theory of planned behavior. Frontiers in Psychology, 13, 1008705. 10.3389/fpsyg.2022.1008705 36562051 PMC9764080

[brb33584-bib-0073] Phuc, N. T. , Giang, N. T. H. , An, V. N. T. T. , Nam, N. T. H. , Anh, L. D. , Nguyen, H. C. , & Hieu, N. H. (2023). Optimization of the eco‐friendly synthesis of graphene oxide from graphite using Plackett–Burman and Box–Behnken models for industrial production orientation. Carbon Letters, 33(2), 489–500. 10.1007/s42823-022-00439-2

[brb33584-bib-0074] PrakashYadav, G. , & Rai, J. (2017). The generation Z and their social media usage: A review and a research outline. Global Journal of Enterprise Information System, 9(2), 110–116. 10.18311/gjeis/2017/15748

[brb33584-bib-0075] Pratama, A. A. N. , Hamidi, M. L. , & Cahyono, E. (2023). The effect of halal brand awareness on purchase intention Indonesiasia: The mediating role of attitude. Cogent Business & Management, 10(1), 2168510. 10.1080/23311975.2023.2168510

[brb33584-bib-0076] Qazi, H. , Alam, S. , & Phulpoto, A. (2023). Motivating factors influencing green buying behavior of generation Z: An application of theory of planned behavior (TPB). Pakistan Journal of Humanities and Social Sciences, 11(3), 3649–3665. 10.52131/pjhss.2023.1103.0646

[brb33584-bib-0077] Rahmadhani, A. V. , & Widodo, A. (2023). Pengaruh green brand image, green brand trust, green brand awareness dan green brand satisfaction terhadap purchase intention pada konsumen air mineral merek aqua. Jurnal Samudra Ekonomi dan Bisnis, 14(3), 393–405. 10.33059/jseb.v14i3.3891

[brb33584-bib-0078] Serafini, K. , Malin‐Mayor, B. , Nich, C. , Hunkele, K. , & Carroll, K. M. (2016). Psychometric properties of the Positive and Negative Affect Schedule (PANAS) in a heterogeneous sample of substance users. The American Journal of Drug and Alcohol Abuse, 42(2), 203–212. 10.3109/00952990.2015.1133632 26905228 PMC4834560

[brb33584-bib-0079] Sharma, A. P. (2021). Consumers’ purchase behavior and green marketing: A synthesis, review and agenda. International Journal of Consumer Studies, 45(6), 1217–1238. 10.1111/ijcs.12722

[brb33584-bib-0080] Shukla, S. (2019). A study on millennial purchase intention of green products in India: Applying extended theory of planned behavior model. Journal of Asia‐Pacific Business, 20(4), 322–350. 10.1080/10599231.2019.1684171

[brb33584-bib-0081] Silvi, M. , & Padilla, E. (2021). Pro‐environmental behavior: Social norms, intrinsic motivation and external conditions. Environmental Policy and Governance, 31(6), 619–632. 10.1002/eet.1960

[brb33584-bib-0082] Singh, D. , & Kunja, S. R. (2023). Examining the mediating role of brand trust and brand commitment in fostering consumer perceptions toward recycled products. Business Strategy & Development, 6(3), 420–429. 10.1002/bsd2.248

[brb33584-bib-0083] Singh, V. , Kathuria, S. , Puri, D. , & Kapoor, B. (2023). Corporate social responsibility and behavioral intentions: A mediating mechanism of brand recognition. Corporate Social Responsibility and Environmental Management, 30(4), 1698–1711. 10.1002/csr.2445

[brb33584-bib-0084] Siyal, S. , Ahmed, M. J. , Ahmad, R. , Khan, B. S. , & Xin, C. (2021). Factors influencing green purchase intention: Moderating role of green brand knowledge. International Journal of Environmental Research and Public Health, 18(20), 10762. 10.3390/ijerph182010762 34682507 PMC8535627

[brb33584-bib-0085] Sousa, S. , Correia, E. , Viseu, C. , & Larguinho, M. (2022). Analyzing the influence of companies’ green communication in college students’ green purchase behavior: An application of the extended theory of planned behavior model. Administrative Sciences, 12(3), 80. 10.3390/admsci12030080

[brb33584-bib-0086] Sun, Y. , Luo, B. , Wang, S. , & Fang, W. (2021). What you see is meaningful: Does green advertising change the intentions of consumers to purchase eco‐labeled products? Business Strategy and the Environment, 30(1), 694–704. 10.1002/bse.2648

[brb33584-bib-0087] Sun, Y. , Wang, R. , Cao, D. , & Lee, R. (2022). Who are social media influencers for luxury fashion consumption of the Chinese Gen Z? Categorisation and empirical examination. Journal of Fashion Marketing and Management: An International Journal, 26(4), 603–621. 10.1108/JFMM-07-2020-0132

[brb33584-bib-0088] Tan, Y. , Geng, S. , Katsumata, S. , & Xiong, X. (2021). The effects of ad heuristic and systematic cues on consumer brand awareness and purchase intention: Investigating the bias effect of heuristic information processing. Journal of Retailing and Consumer Services, 63, 102696. 10.1016/j.jretconser.2021.102696

[brb33584-bib-0089] Tan, Z. , Sadiq, B. , Bashir, T. , Mahmood, H. , & Rasool, Y. (2022). Investigating the impact of green marketing components on purchase intention: The mediating role of brand image and brand trust. Sustainability, 14(10), 5939. 10.3390/su14105939

[brb33584-bib-0090] Tsendsuren, C. , Yadav, P. L. , Han, S. H. , & Kim, H. (2021). Influence of product market competition and managerial competency on corporate environmental responsibility: Evidence from the US. Journal of Cleaner Production, 304, 127065. 10.1016/j.jclepro.2021.127065

[brb33584-bib-0091] United Nations (UN) . (1987). Report of the world commission on environment and development: Our common future . United Nations. https://sustainabledevelopment.un.org/content/documents/5987our‐common‐future.pdf

[brb33584-bib-0092] United Nations (UN) . (2024). Department of economic and social affairs sustainable development, “The 17 Goals” . United Nations. https://sdgs.un.org/goals

[brb33584-bib-0093] Uyanah, D. A. , & Nsikhe, U. I. (2023). The theoretical and empirical equivalence of Cronbach alpha and Kuder‐Richardson formular‐20 reliability coefficients. International Research Journal of Innovations in Engineering and Technology, 7(5), 17. 10.47001/IRJIET/2023.705003

[brb33584-bib-0094] Vasan, M. (2023). Impact of promotional marketing using Web 2.0 tools on purchase decision of Gen Z. Materials Today: Proceedings, 81, 273–276. 10.1016/j.matpr.2021.03.188

[brb33584-bib-0095] Walker, O. C. Jr. , & Ruekert, R. W. (1987). Marketing's role in the implementation of business strategies: A critical review and conceptual framework. Journal of Marketing, 51(3), 15–33. 10.1177/002224298705100302

[brb33584-bib-0096] Wang, L. , Zhang, Q. , & Wong, P. P. W. (2022). Purchase intention for green cars among Chinese millennials: Merging the value–attitude–behavior theory and theory of planned behavior. Frontiers in Psychology, 13, 786292. 10.3389/fpsyg.2022.786292 35273539 PMC8902249

[brb33584-bib-0097] Wang, Q. C. , Lou, Y. N. , Liu, X. , Jin, X. , Li, X. , & Xu, Q. (2023). Determinants and mechanisms driving energy‐saving behaviours of long‐stay hotel guests: Comparison of leisure, business and extended‐stay residential cases. Energy Reports, 9, 1354–1365. 10.1016/j.egyr.2022.12.051

[brb33584-bib-0098] Wang, Y. M. , Zaman, H. M. F. , & Alvi, A. K. (2022). Linkage of green brand positioning and green customer value with green purchase intention: The mediating and moderating role of attitude toward green brand and green trust. Sage Open, 12(2), 21582440221102441. 10.1177/21582440221102441

[brb33584-bib-0099] Watson, A. , Perrigot, R. , & Dada, O. (2024). The effects of green brand image on brand loyalty: The case of mainstream fast food brands. Business Strategy and the Environment, 33(2), 806–819. 10.1002/bse.3523

[brb33584-bib-0100] Wei, J. , Zhao, X. , & Yang, X. (2021). Measuring purchase intention toward green power certificate in a developing nation: Applying and extending the theory of planned behavior. Resources, Conservation and Recycling, 168, 105363. 10.1016/j.resconrec.2020.105363

[brb33584-bib-0101] Welford, R. (2000). Hijacking environmentalism. Earthscan.

[brb33584-bib-0102] Wiedmer, T. (2015). Generations do differ: Best practices in leading traditionalists, boomers, and generations X, Y, and Z. Delta Kappa Gamma Bulletin, 82(1), 51–58.

[brb33584-bib-0103] Wongsaichia, S. , Naruetharadhol, P. , Schrank, J. , Phoomsom, P. , Sirisoonthonkul, K. , Paiyasen, V. , & Ketkaew, C. (2022). Influences of green eating behaviors underlying the extended theory of planned behavior: A study of market segmentation and purchase intention. Sustainability, 14(13), 8050. 10.3390/su14138050

[brb33584-bib-0104] Wu, L. , Zhu, Y. , & Zhai, J. (2022). Understanding waste management behavior among university students in China: Environmental knowledge, personal norms, and the theory of planned behavior. Frontiers in Psychology, 12, 771723. 10.3389/fpsyg.2021.771723 35095656 PMC8789738

[brb33584-bib-0105] Yoo, B. , & Donthu, N. (2001). Developing and validating a multidimensional consumer‐based brand equity scale. Journal of Business Research, 52(1), 1–14. 10.1016/S0148-2963(99)00098-3

[brb33584-bib-0106] Zeithaml, C. , & Zeithaml, V. (1984). Environmental management: Revising the marketing perspective. Journal of Marketing, 48, 46–53. 10.1177/002224298404800204

[brb33584-bib-0107] Zhou, Z. , Zheng, F. , Lin, J. , & Zhou, N. (2021). The interplay among green brand knowledge, expected eudaimonic well‐being and environmental consciousness on green brand purchase intention. Corporate Social Responsibility and Environmental Management, 28(2), 630–639. 10.1002/csr.2075

